# Neurological adverse events in patients receiving anti-TNF therapy: a prospective imaging and electrophysiological study

**DOI:** 10.1186/ar4582

**Published:** 2014-06-17

**Authors:** Evripidis Kaltsonoudis, Anastasia K Zikou, Paraskevi V Voulgari, Spyridon Konitsiotis, Maria I Argyropoulou, Alexandros A Drosos

**Affiliations:** 1 Rheumatology Clinic, Department of Internal Medicine, Medical School, University of Ioannina, Ioannina, Greece; 2 Department of Clinical Imaging and Radiology, Medical School, University of Ioannina, Ioannina, Greece; 3 Neurology Clinic, Medical School, University of Ioannina, Ioannina, Greece

## Abstract

**Introduction:**

The aim was to investigate the frequency of neurological adverse events in patients with rheumatoid arthritis (RA) and spondylarthropathies (SpA) treated with tumor necrosis factor (TNF) α antagonists.

**Methods:**

Seventy-seven patients eligible for anti-TNFα therapy were evaluated. There were 36 patients with RA, 41 with SpA [24 psoriatic arthritis (PsA) and 17 with ankylosing spondylitis (AS)]. All patients had a complete physical and neurological examination. Brain and cervical spine magnetic resonance imaging (MRI) and neurophysiological tests were performed in all patients before the initiation of anti-TNFα therapy and after a mean of 18 months or when clinical symptoms and signs indicated a neurological disease. Exclusion criteria included hypertension, diabetes mellitus, dyslipidemia, heart arrhythmias, atherothrombotic events, vitamin B12 and iron deficiency, head and neck trauma and neurological surgeries.

**Results:**

Two patients did not receive anti-TNFα therapy because brain MRIs at baseline revealed lesions compatible with demyelinating diseases. Thus, 75 patients received anti-TNFα (38 infliximab, 19 adalimumab and 18 etanercept). Three patients developed neurological adverse events. A 35-year-old man with PsA after 8 months of infliximab therapy presented with paresis of the left facial nerve and brain MRI showed demyelinating lesions. Infliximab was discontinued and he was treated with pulses of corticosteroids recovering completely after two months. The second patient was a 45-year-old woman with RA who after 6 months of adalimumab therapy presented with optic neuritis. The third patient was a 50-year-old woman with AS, whom after 25 months of infliximab therapy, presented with tingling and numbness of the lower extremities and neurophysiological tests revealed peripheral neuropathy. In both patients anti-TNF were discontinued and they improved without treatment after 2 months. The rest of our patients showed no symptoms and MRIs showed no abnormalities. The estimated rate of neurological adverse events in patients treated with anti-TNF therapy is 4% (3/75).

**Conclusions:**

Neurological adverse events after anti-TNFα therapy were observed in our patient. Brain MRI and neurophysiological tests are essential tools to discriminate neurological diseases.

## Introduction

TNFα antagonists are a significant advantage for the treatment of rheumatoid arthritis (RA), spondyloarthropathies (SpA), and other inflammatory diseases [1]. These agents have proven to be more effective than traditional disease-modifying antirheumatic drugs (DMARDs) and may prevent development of structural damage [2-8]. However, their increasing use during the last decade has revealed a variety of immune-mediated adverse events [9]. Clinical signs of autoimmune diseases, such as urticaria, psoriasis, lupus-like syndrome, diabetes mellitus type I and others have been reported [10-13]. In addition, numerous reports and case series of neurological adverse events due to anti-TNFα blockers have been reported. These include demyelinating conditions, optic neuritis, chronic inflammatory demyelinating polyneuropathy, mononeuritis multiplex, Guillain-Barré syndrome and others [14-41]. However, there is debate about whether treatment with anti-TNFα blockers unmasks preexisting demyelinating disorders such as multiple sclerosis (MS) or induces *de novo* demyelination of the central nervous system (CNS) and peripheral nervous system. On the other hand, patients with RA and SpA may develop neurological manifestations mostly due to cervical spine involvement and CNS disease due to vasculitis or amyloidosis [42,43]. In addition patients with RA may develop peripheral nervous system involvement such as sensorimotor neuropathy or mononeuritis multiplex (42).

To date, only case reports and case series have been reported. For this reason we undertook a prospective study using magnetic resonance imaging (MRI) and neurophysiological tests in patients with RA and SpA receiving anti-TNFα antagonists.

## Materials and methods

Patients with RA and SpA who were followed up at a single tertiary Rheumatology center, and who were eligible for anti-TNFα treatment between May 2009 and December 2011 were included. Patients with RA fulfilled the American College of Rheumatology (ACR) 1987 for the disease [44] and patients with SpA fulfilled the Assessment of SpondyloArthritis international Society (ASAS) criteria [45]. Exclusion criteria included severe uncontrolled hypertension, diabetes mellitus, dyslipidemia, atherothrombotic events, heart arrhythmias, vitamin B12 and iron deficiency, as well as head and neck trauma, neurological surgery or any other neurological conditions. All patients underwent complete physical examination and detailed neurological evaluation which included also brain and cervical spine MRI as well as neurophysiology testing with nerve conduction velocity and needle electromyography (EMG). Neurological evaluation and neurophysiologial tests were performed by an expert neurologist (SK), who was unaware of patient history. Patients received anti-TNFα therapy and were followed up every 2 to 3 months with appropriate laboratory monitoring, as well as with complete physical examination. MRI and neurophysiology testing were repeated after a mean period of 18 months after treatment or when clinical symptoms and signs indicated neurological disease.

All MRI was performed on the same 1.5 T unit (Gyroscan Intera; Philips Healthcare, Best, The Netherlands) by using a quadrature head coil. The imaging protocol consisted of: (i) T1-weighted high resolution (1 × 1 × 1 mm) three-dimensional spoiled gradient-echo sequence (repetition time (TR), 25 ms; echo time (TE), 4.6 ms; acquisition matrix, 256 × 228; field of view (FOV), 220 mm; number of signal intensity averages, 1), which was used for structural imaging; (ii) axial T2-weighted sequence (TR, 3,000 ms; TE, 90 ms; FOV, 250 mm; matrix, 276 × 176; section thickness, 6 mm; number of signal intensity averages, 2; intersection gap, 0.6; and (iii) a sagittal fluid attenuated inversion recovery (FLAIR) sequence (TR, 6,300 ms; TE, 120 ms; FOV: 250 mm; matrix, 256 × 256; section thickness, 6 mm; intersection gap, 0.6; number of signal intensity averages, 2), which was used for evaluation of white-matter hyper-intensity. Study subjects' informed consent and approval from the institutional ethical committee (University Hospital of Ioannina) were obtained. All MRI scans were read by two expert radiologists (AKZ) and (MIA) who were also unaware of the patients' history.

Finally, all patients had an immunologic evaluation before initiating anti-TNFα therapy, which included antinuclear antibodies (ANA), antibodies to double-stranded DNA, anticardiolipin antibodies (ACL), and antibodies to β_2_GPI as well as lupus anticoagulant (LA).

## Results

**Figure 1:**
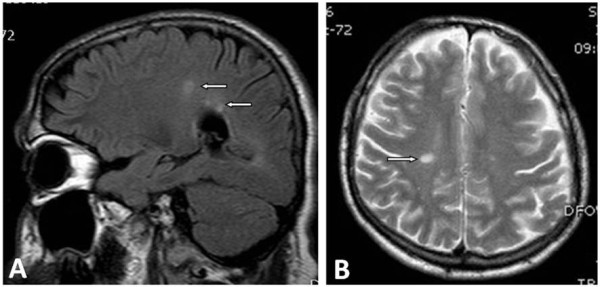
Sagittal fluid attenuated inversion recovery (FLAIR) (A) and axial T2-weighted (B) scans demonstrating ovoid hyperintense lesions in the deep periventricular white matter (arrows).

**Figure 2:**
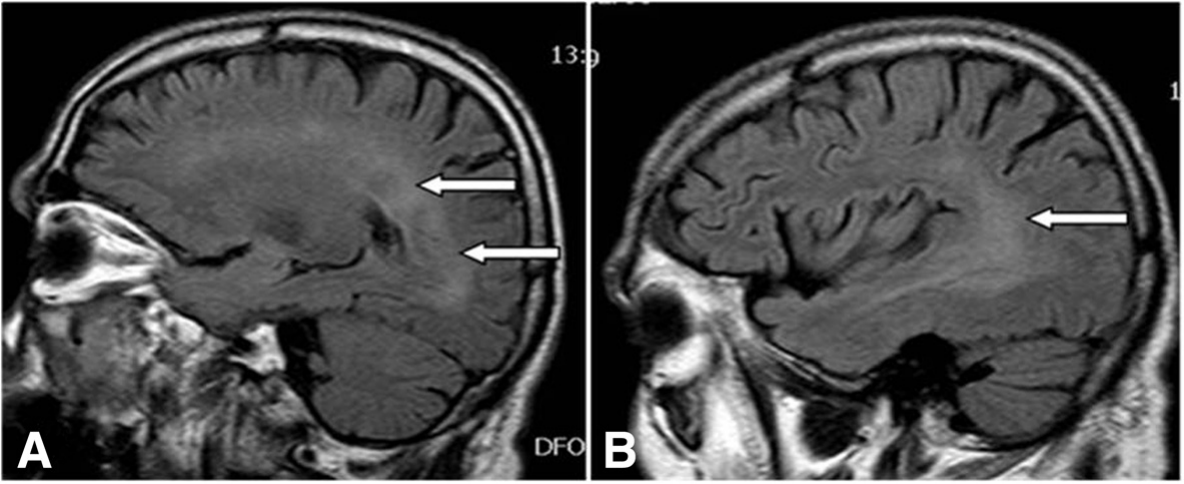
Sagittal fluid attenuated inversion recovery (FLAIR) scans (A, B) show bilateral diffuse hyperintense signal in the periventricular white matter of the parietal, occipital and temporal lobes (arrows).

In total 101 patients were evaluated; 24 patients were excluded because 10 of them had hypertension and dyslipidemia, 5 had diabetes mellitus, 4 had atrial fibrillation, 1 had vitamin B12 deficiency and 4 refused to undergo MRI and EMG. Thus, 77 patients were included. Of these, 36 patients had RA and 41 SpA (24 with psoriatic arthritis (PsA) and 17 with ankylosing spondylitis (AS)). The demographic characteristics are depicted in the table. Before the initiation of anti-TNFα therapy, one patient with AS reported numbness of the left hand and dizziness. Neurological examination, head and cervical spine MRI and neurophysiology testing revealed no abnormalities. However, two patients, a 35-year-old man with AS and a 46-year-old woman with PsA, did not receive anti-TNFα therapy because brain MRI revealed lesions compatible with or suggestive of demyelinating disease (Figures 1 and 2).

A total of 75 patients received a first anti-TNFα therapy except one patient with PsA who had switched from etanercept to infliximab due to inadequate response after 10 months of treatment. Thus, 38 patients received infliximab, 19 adalimumab and 18 etanercept with or without DMARDs and/or a small dose of steroids (Table 1). Patients were followed up for an average period of 18 months, ranging from 16 to 26 months. During this period, three patients experienced neurological complications, while the rest of the patients had no neurological symptoms, and results of MRI and neurophysiological tests were within normal limits. One patient with SpA who had partially responded to etanercept was switched to infliximab. All other patients responded well to initial anti-TNFα therapy.

**Table 1:** Demographic characteristics of patients eligible for anti-TNF therapy

**Variable**	**Value**
Patients, n	**77**
Male/female, n	42/35
Average age, years, n (SD)	55.3 (12.5)
Rheumatoid arthritis patients, n (%)	36 (46.8)
Psoriatic arthritis patients, n (%)	24 (31.2)
Ankylosing spondylitis patients, n (%)	17 (22.1)
DMARDs intake, n (%)	55 (71.4)
Methotrexate, n (%)	31 (56.4)
Cyclosporin, n (%)	10 (18.0)
Leflunomide, n (%)	4 (7.0)
Steroids intake, n (%)	12 (15.6)
Anti-TNF intake, n (%)	75 (97.4)
Infliximab, n (%)	38 (51.0)
Adalimumab, n (%)	19 (25.0)
Etanercept, n (%)	18 (24.0)
Antinuclear antibodies positivity, n (%)*	10 (13.0)
Rheumatic factor positivity, n (%)*	28 (36.4)
Anti-citrullinated antibodies positivity, n (%)*	29 (37.7)

*Refers to the total number of the patients in this study.

Examples of specific cases are as follows.

### Case 1

**Figure 3:**
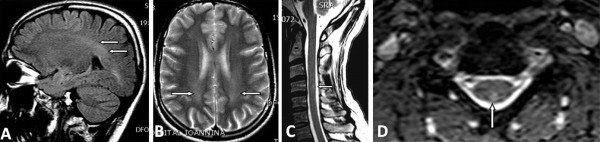
**Magnetic resonance imaging (MRI) lesions compatible with demyelinating disease.** Sagittal fluid attenuated inversion recovery **(A)** and axial T2-weighted **(B)** scans show bilateral diffuse hyperintense signal in the periventricular white matter of the parietal and occipital lobes (arrows). T2-weighted sagittal **(C)** and axial **(D)** spinal cord scans reveal a small peripherally located lesion (arrows).

A 35-year-old man with PsA, who was initially treated with etanercept and then switched to infliximab, presented after 8 months of infliximab therapy with difficulty in speech, swallowing and ptosis of the left corner of the mouth. He also experienced tingling and numbness of the left lower extremity. Neurological examination revealed peripheral paresis of the left facial nerve, and unilateral peroneal nerve palsy. Routine laboratory tests were unremarkable and ANA, ACL, β_2_GPI and LA were negative. Brain and spinal cord MRI revealed lesions compatible with demyelinating disease (Figure 3). Neurophysiology testing of the lower extremities showed findings of left peroneal nerve lesion with active denervation on EMG. The patient declined examination of the cerebrospinal fluid (CSF). Infliximab was discontinued and he was treated with intravenous boluses of methyl-prednisone (1 g/day for 5 days) with significant clinical improvement. The full restoration of neurological symptoms occurred after two months of infliximab discontinuation.

### Case 2

A 45-year-old woman with RA after 6 months of adalimumab treatment presented with a reduction of visual acuity on the right eye, and ocular pain mainly on eye movements. Neurological examination was normal. Fundoscopic examination showed no abnormalities. A diagnosis of right-sided optic neuritis was done and adalimumab was discontinued. Brain MRI revealed no abnormalities and the patient recovered gradually without treatment. After 2 months the patient had a complete recovery. However, the patient had a flare of RA symptoms; therefore adalimumab was restarted after informed consent. A second episode of optic neuritis this time in the left eye was manifested after two adalimumab injections and the drug was stopped permanently. A new brain MRI revealed no abnormalities. The patient declined CSF tap and she had a close follow up without treatment. Results of laboratory and immunology tests (ANA, ACL, β_2_GPI, LA) were within normal limits or negative. She recovered completely after 2 months.

### Case 3

A 50-year-old woman suffering from AS and Crohn’s disease was treated with infliximab with significant improvement of both diseases. After 25 months of infliximab therapy, she presented with tingling and numbness affecting both legs. Neurological examination revealed bilateral decrease of deep tendon reflexes of the lower limbs as well as hypoesthesia below the knees. Brain MRI showed no pathological findings, while neurophysiology testing revealed a significant reduction in sensory nerve conduction velocity in the lower extremities. Infliximab was discontinued and gabapentin 1,600 mg/day was started, with partial improvement of the sensory symptoms. After 3 months of infliximab discontinuation, and after the patient’s informed consent, infliximab was re-administered. However, after the first re-infusion, neurological symptoms (tingling, numbness and dysesthesias) reappeared in the lower extremities and the drug was discontinued. Routine laboratory and immunological evaluation (ANA, ACL, β_2_GPI, LA) showed no abnormalities.

## Discussion

We investigated the characteristics and frequency of neurological adverse events in patients with rheumatic diseases treated with TNFα antagonists in a prospective study using MRI and neurophysiological tests before the initiation of anti-TNFα therapy. We described three cases with neurological adverse events, the first showing CNS demyelinating disease and peripheral neuropathy, the second with recurrent optic neuritis and the third with peripheral neuropathy. TNFα blockers are known to potentially cause neurological adverse events, which may be part of an MS-like syndrome or represent new inflammatory demyelinating disease. However, there is a question as to whether this association actually represents an unmasking of preexisting, presymptomatic MS or the onset of new demyelinating disease.

Interestingly, using MRI we identified two patients before the initiation of anti-TNFα therapy as having features of demyelination in the CNS. These two patients never received anti-TNF blockers and no neurological symptoms had developed after 2 years of follow up. On the other hand, if these two patients had been treated with TNFα antagonists without having MRI at baseline, they might have developed symptoms and signs of demyelinating disease, but equally they may not have. These two patients represent a population of patients with a preexisting, asymptomatic demyelinating disease, which may become manifest after anti-TNFα therapy. In this situation, the preexisting evaluation with MRI is highly important.

Unexpected incidental MRI findings suggestive of MS without typical MS symptoms, is now a well-recognized condition known as radiologically isolated syndrome (RIS). This has led to an increased awareness of this condition and studies by several groups have shown that there is a close association between RIS and MS, such that in some cases RIS may be considered to be preclinical MS [46].

Three more patients developed neurological adverse events after anti-TNFα treatment. These cases were new-onset diseases (CNS demyelination, optic neuritis and peripheral neuropathy). The question arises as to how this happened.

The mechanisms underlying the predisposition to demyelination or exacerbation of demyelination in patients treated with TNFα antagonists is not well-understood. Several hypotheses have been proposed [47]. TNFα has a critical role in MS. It is clearly a pro-inflammatory cytokine during the acute phase of the disease and participates in the demyelinating process. On the other hand, TNFα also has immunosuppressive properties during the later phase of the disease. These properties are related to TNF receptors (TNFR)1 and 2, which mediate differential biological responses of TNFα. Within CNS, TNFα is produced by microglia, astrocytes or other cells as a monomeric transmembrane precursor protein (tmTNF). The cytoplasmic tail is then cleaved by the TNFα converting enzyme, releasing the soluble form of TNFα (sTNFα). To perform their biological function, tmTNF and sTNF monomerics must aggregate to form homotrimers. Both TNFα (sTNF and tmTNF) can bind to both TNFR1 and TNFR2; sTNFα shows greater affinity for TNFR1 than TNFR2 causing inflammatory responses, leading to apoptosis, while tmTNFα acts mainly on TNFR2 leading to activation and cell survival. Isolated expression of the tmTNF in the transgenic mice can suppress the initiation and progression of experimental autoimmune encephalitis (EAE) and at the same time maintains the properties of autotolerance and resistance to infection. Thus, selective inhibition of sTNF/TNFR1 signaling might be used as a therapeutic strategy for prevention or relapsing MS [47].

Several theories have been proposed to explain a potential biologic relationship between TNFα antagonists and demyelinating disease: (a) TNFα blockers do not penetrate the blood–brain barrier, but enhance disease activity via an increase in peripheral T-cell autoreactive cells, which can penetrate into CNS [17]; (b) down regulation of TNFR2 necessary for the proliferation of oligodendrocytes and damage repair [17,23]; (c) down regulation of downstream production of cytokines such as IL-10 and up regulation of IL-12 and IFN-γ associated with demyelinating disease process [48,49]; (d) TNFα antagonists could unmask a latent infection critical to inciting an autoimmune demyelinating process [50].

It has been suggested that TNFα antagonists may increase the risk of demyelinating diseases in patients with RA by about 30% [32], however, these data are not supported by others [38]. The overall prevalence of RA and MS is 0.6% and 0.05% respectively [23]. The occurrence of both diseases in the same patient has been reported [51]. This coincidence should be not surprising because both RA and MS share pathogenetic and genetic similarities [51]. In this context patients having one autoimmune disease are at increased risk of developing another. In favor of this, is the existence in our study of two patients with preexisting MS-like lesions.

The development of central and peripheral nervous system demyelinating lesions after the introduction of anti-TNF in three of our patients supports the idea of anti-TNF evoking demyelination. The fact that in two out of three patients the symptoms improved with cessation of anti-TNF and re-emerged with reintroduction of anti-TNF clearly supports the theory of anti-TNF-evoked pathophysiology. Thus, patients who already are at increased risk (due to a genetic predisposition) of developing immune-mediated demyelination, may be at increased risk of developing neurological diseases after the introduction of anti-TNF. In our study, the estimated rate of neurological adverse events in patients with inflammatory arthritides treated with anti-TNF therapy is 4% (3/75). Our results differ somewhat from previous studies. In RA patients, a frequency of 30% of adverse events, mostly peripheral polyneuropathy, has been reported after anti-TNF therapy [32], while other studies have not supported these results [38]. However, our study is the only prospective study in which all patients had neurophysiological tests and MRI before and after anti-TNF therapy. This might explain the different frequency of neurological adverse events found in our study. Therefore, further prospective well-controlled studies are needed to confirm our results.

In summary, the estimated rate of neurological adverse events in patients with rheumatic diseases treated with TNFα antagonists is 4%. Brain MRI and neurophysiology testing are essential tools to discriminate subclinical preexisting demyelinating diseases. In patients who are candidates for anti-TNFα therapy a detail clinical and neurological examination is necessary. Finally, close follow up and appropriate monitoring are essential and when the patients develop symptoms or signs of neurological adverse events, TNFα antagonists should be discontinued and appropriate tests should be performed.

## Conclusion

The development of central and peripheral nervous system adverse events after the introduction of anti-TNF in three of our patients supports the notion of anti-TNF-evoked demyelination.

### Statement

Written informed consent was obtained from the patients for publication of their individual details and accompanying images in this manuscript. The consent form is held by the authors in the patients clinical notes and is available for review by the Editor-in-Chief.

## Abbreviations

ACL: anticardiolipin antibodies; ACR: American College of Rheumatology; ANA: antinuclear antibodies; AS: ankylosing spondylitis; ASAS: Assessment of SpondyloArthritis international Society; CNS: central nervous system; CSF: cerebrospinal fluid; DMARD: disease-modifying antirheumatic drug; EMG: electromyography; FLAIR: fluid attenuated inversion recovery; FOV: field of view; IL: interleukin; LA: lupus anticoagulant; MRI: magnetic resonance imaging; MS: multiple sclerosis; PsA: psoriatic arthritis; RA: rheumatoid arthritis; RIS: radiologically isolated syndrome; SpA: spondylarthropathies; sTNFα: soluble tumor necrosis factor α; TE: echo time; tmTNF: transmembrane precursor protein; TNF: tumor necrosis factor; TNFR: tumor necrosis factor receptor; TR: repetition time.

## Competing interests

The authors declare that they have no competing interests.

## Authors’ contributions

EK: data collection and interpretation, and drafting of manuscript: AKZ: imaging evaluation and data interpretation: PPV: data collection and interpretation, and revision of the manuscript; SK: neurological evaluation and data interpretation: MIA: imaging evaluation and data interpretation: AAD: conception and design, and revision of the manuscript. All authors read and approved the final manuscript.
